# New occurrence of B chromosomes in *Partamonahelleri* (Friese, 1900) (Hymenoptera, Meliponini)

**DOI:** 10.1590/S1415-47572009005000065

**Published:** 2009-12-01

**Authors:** Cinthia Caroline Cardoso Martins, Olivia Maria Pereira Duarte, Ana Maria Waldschmidt, Rogério Marco de Oliveira Alves, Marco Antônio Costa

**Affiliations:** 1 Universidade Estadual de Santa Cruz, Departamento de Ciências Biológicas, Ilhéus, BABrazil; 2 Departamento de Ciências Biológicas, Universidade Estadual do Sudoeste da Bahia, Jequié, BABrazil; 3 Universidade Federal do Recôncavo Baiano, Cruz das Almas, BahiaBrazil

**Keywords:** stingless Bee, *Partamona helleri*, geographic variation, supernumerary chromosomes

## Abstract

Cytogenetic analyses of the stingless bee *Partamona helleri* collected in the state of Bahia, Northeast Brazil revealed the chromosome numbers n = 18 in the haploid males and 2n = 35 in the diploid females. All karyotypes displayed one large acrocentric B chromosome, which differs from the minute B chromosomes previously described in the populations from southeastern Brazil. Giemsa staining, C-banding and DAPI/CMA_3_ fluorochrome staining also revealed a remarkable interpopulational divergence regarding both the regular karyotype and the B chromosomes. The B chromosomes found in the samples from Jequié, Bahia, were entirely heterochromatic, while those found in Cravolândia, Bahia, displayed a euchromatic portion at the telomeric end of the long arm. CMA _3_ labeling sites varied from seven to eight between the two localities in Bahia, due to the presence of an extra GC-rich block in the karyotype of the samples from Jequié. This is the first report of a large B chromosome in *P. helleri* and reveals the occurrence of a geographic differentiation within this species.

*Partamona* is a Neotropical genus of stingless bees with an ample distribution in a wide variety of habitats going from southern Brazil to central Mexico. These habitats include rain forests, cerrado (Brazilian savanna), caatinga, and highlands. Given the existence of morphologically similar species, this group has a problematic taxonomy. Some species can only be recognized by their nesting behavior or nest entrance architecture (Pedro and Camargo, 2003).

Of the 33 species currently recognized (Pedro and Camargo, 2003), only eight, *P. pearsoni* (Tarelho ZVS, 1973, MSc Dissertation, University of São Paulo), *P. seridoensis* (Brito-Ribon *et al.*, 1999, 2005;), *P. aiylae*, *P. vicina*, *P. mulata, P. nhambiquara* (Brito-Ribon *et al.*, 1999), *P. peckolti* (Brito *et al.*, 2003), and *P. helleri* (Costa *et al.*, 1992; Brito *et al.*, 1997, 2005), were cytogenetically studied. All these species showed the regular chromosome number 2n = 34, but *P. helleri* showed a diploid numeric variation ranging from 2n = 34 to 2n = 38, due to the occurrence of up to four minute B chromosomes per individual (Costa *et al.*, 1992; Brito *et al.*, 1997, 2005; Tosta *et al.*, 2004).

B chromosomes are extra chromosomes to the regular complement and are characterized by their dispensability, independent evolution, and non-Mendelian patterns of inheritance (Beukeboom, 1994). Their origin is a matter of recurrent debate among cytologists. Some have proposed that the appearance of the B chromosomes suggests the involvement of rearrangements in the regular chromosomes, *e.g.*, centric fragment formation through chromosome fusions (Camacho *et al.*, 2000). An alternative hypothesis suggests an origin through interspecific hybridization as observed in the fish *Poecilia formosa* (Schartl *et al.*, 1995) and in the wasp *Nasonia vitripennis* (McAllister and Werren, 1997).

Brito *et al.* (1997) distinguished two morphological types of B chromosomes occurring in *P. helleri* in southeastern Brazil. Although the presence of the minute B's was common in the previously studied population, the fourfold B dosage [2n = 38 chromosomes, Tosta *et al.* (2004)] was rare. This finding suggests the existence of a mechanism controlling or preventing the accumulation of B chromosomes in individuals.

Recently molecular studies have been started, in an attempt to better understand the population dynamics of B chromosomes in *P. helleri.* Tosta *et al.* (2007) developed a SCAR (Sequence Characterized Amplified Region) marker potentially useful for analyzing the frequency, geographic distribution, transmission, or effects of the B chromosome in the adult organism. However, despite the contributions given by previous studies, the origin of the B's in *P. helleri* is so far still unclear.

Here, we report a new type of B chromosome in populations from the northern limits of the distribution of *P. helleri* in the state of Bahia, Brazil. Differences from previous descriptions found in the regular karyotype are also discussed.

One nest of *P. helleri* from Jequié (13° 52' S, 40° 13' W) and one nest from Cravolândia (13° 21' S, 39° 48' W), both in the state of Bahia, Northeast Brazil, were collected for cytogenetic analyses.

Metaphases were obtained from cerebral ganglia of prepupae, following the protocol described by Imai *et al.* (1988). A total of 50 specimens were analyzed.

We performed C banding according to Sumner (1972), with the slight modifications proposed by Pompolo and Takahashi (1990). DAPI/CMA_3_ staining was done as described by Schweizer (1980).

C-banded and conventional Giemsa-stained chromosomes were analyzed and photographed with a Leica DLMS photomicroscope using HQ film. Fluorescent images were captured in a Leica DMRA2 photomicroscope using the IM50 software. In order to compare our results with previous studies, the karyotypes were arranged by decreasing order of length of euchromatic arms, and the chromosome nomenclature followed Imai (1991), with metacentric chromosomes showing pericentomeric (M^cc^), centromeric (M^c^), pericentromeric and telomeric (M^cct^), centromeric and telomeric (M^ct^), and pericentromeric and interstitial (M^cci^) heterochromatic bands (see the original reference for more details).

In the present analyses, the karyotype of *P. helleri* showed 35 chromosomes in the females and 18 chromosomes in the males. For the Cravolândia samples, the C-banded diploid karyotypic formula found was 2k = 28M^cc^+ 4M^c^+ 2M^cct^+1B (Figure 1d).

**Figure 1:**
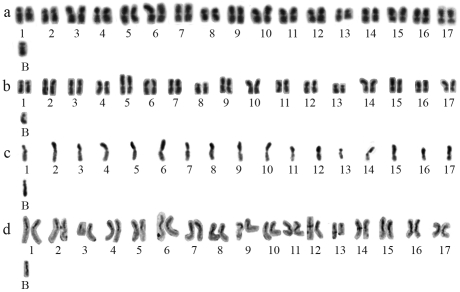
*P. helleri* karyotypes with one B chromosome of: a) a female (2n = 35) from Jequié, b) a female (2n = 35) from Cravolândia after Giemsa staining, c) a male (n = 18) from Jequié, and d) a female (2n = 35) from Cravolândia after C-banding.

Previous studies had reported the karyotypic formula 2k = 20M^cc^+4M+8M^ct^+2M^cci^ + 1B for the 2n = 35 karyotypes sampled in the state of Minas Gerais (Brito *et al.*, 1997, 2005). In our study, metacentric chromosomes with pericentromeric and interstitial heterochromatin (M^cci^) were not observed.

The novel B chromosomes found in the Bahia samples were much larger than those described in the population from southeastern Brazil (Brito *et al.*, 1997, 2005). They had an acrocentric morphology and a size comparable to the chromosomes of the regular complement. The B chromosomes observed in the Jequié samples were entirely heterochromatic, whereas those found in Cravolândia showed a euchromatic portion at the telomeric end of the long arm (Figures 1c, d). These results reveal a substantial karyotypic divergence among the populations studied so far, due to structural changes in both the regular and the B chromosomes.

Brito *et al.* (1997) classified the minute B's found in the southeastern Brazilian populations as B1 (heterochromatic submetacentrics) and B2 (acrocentrics, with C-banding undetermined due to their small size). Partially and entirely heterochromatic B chromosomes were found in another bee species, *Meliponaquinquefasciata* (Marla P. Rocha, personal communication).

The DAPI fluorochrome stained the heterochromatin in all centromeric and pericentromeric regions of regular chromosomes (Figures 2b, c), but the CMA_3_ fluorochrome staining was variable. Specimens from Jequié showed eight CMA_3_^+^ labeling sites, seven on the regular chromosomes and one on the B chromosome (Figure 2a). In the samples from Cravolândia, only seven CMA_3_ labeling sites were observed, six on the regular chromosomes and one on the B chromosome (Figures 2c). The latter result agrees with Brito *et al.* (2005), who also found seven CMA_3_^+^ markings on regular chromosomes and one on the minute B in *P. helleri* from southeastern Brazil. These authors also found a heteromorphism in the second chromosome pair due to a difference in the size of the heterochromatic/CMA_3_^+^ long arm, a result not observed in the Bahia populations. Similar heterogeneous AT- and GC-rich heterochromatin blocks were observed in other meliponine species, such as *Plebeia* sp*.* and *Melipona* sp*.* (Maffei *et al.*, 2001), and *Partamona peckolti* (Brito *et al.*, 2003).

The present analysis using different techniques revealed the occurrence of a geographic differentiation between the populations of *P. helleri*. We observed karyotypic differences in the size, morphology, and distribution of the heterochromatin in the chromosomes of the regular complement and of the B chromosomes. Our results show that the B chromosome system of *P. helleri* is more complex than previously assumed. The large B chromosomes found in the Brazilian Northeast region can be an important element for future evolutionary studies of this species.

**Figure 2:**
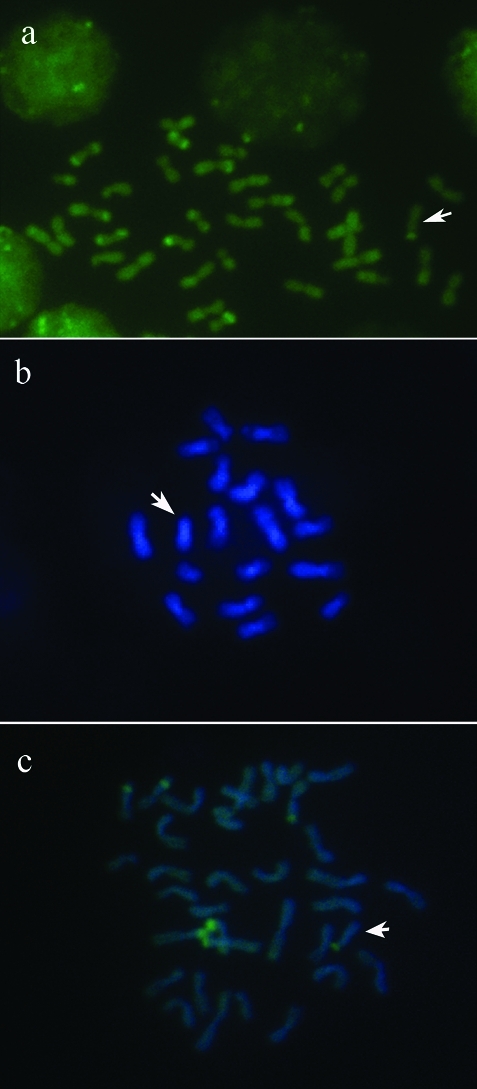
Partial exon II sequence of the *SERPINA1* gene (cloned DNA) reveals the presence of c.97 T → C and c.302 G → A substitutions on the same chromosome.

In the study carried out by Tosta *et al.* (2007), the authors found a SCAR (Sequence Characterized Amplified Region) marker associated with the minute B chromosomes of animals from the southeastern region. This was the first attempt to use molecular data to investigate the effect of B chromosomes on the individuals and showed that the approach could be effective for future population studies of this species, especially concerning the origin of the B's.

The karyotypic differences described indicate that the B chromosomes found in the present study and those previously reported in the southeastern region could have had distinct origins, probably involving rearrangements in several of the regular chromosomes. The so far undefined origins and patterns of the B chromosome inheritance can be clarified with an ample analysis of its variation and geographic distribution in this species. Other approaches, including molecular and cytogenetic data, will help to clarify the cytotaxonomy and the mechanisms involved in the karyotype evolution of *P*. *helleri.* We have already been carrying out new samplings in order to further characterize the cytogenetic and molecular diversity of this species, especially in Bahia where the large Bs were found.
